# Detection and Potential Virulence of Viable but Non-Culturable (VBNC) *Listeria monocytogenes*: A Review

**DOI:** 10.3390/microorganisms9010194

**Published:** 2021-01-19

**Authors:** Nathan E. Wideman, James D. Oliver, Philip Glen Crandall, Nathan A. Jarvis

**Affiliations:** 1 Corporate Laboratory, Tyson Foods, 3609 Johnson Rd, Springdale, AR 72762, USA; nwideman@uark.edu; 2 Department of Biological Sciences, UNC Charlotte, Charlotte, NC 28223, USA; jdoliver@uncc.edu; 3 Department of Food Science and Center for Food Safety, University of Arkansas, 2650 N. Young Ave., Fayetteville, AR 72704, USA; 4 Conrad N. Hilton College of Hotel and Restaurant Management, University of Houston, 122 Heiman Street, San Antonio, TX 78205, USA; najarvis@uh.edu

**Keywords:** *Listeria monocytogenes*, viable but non-culturable, VBNC, virulence, detection methods

## Abstract

The detection, enumeration, and virulence potential of viable but non-culturable (VBNC) pathogens continues to be a topic of discussion. While there is a lack of definitive evidence that VBNC *Listeria monocytogenes* (Lm) pose a public health risk, recent studies suggest that Lm in its VBNC state remains virulent. VBNC bacteria cannot be enumerated by traditional plating methods, so the results from routine Lm testing may not demonstrate a sample’s true hazard to public health. We suggest that supplementing routine Lm testing methods with methods designed to enumerate VBNC cells may more accurately represent the true level of risk. This review summarizes five methods for enumerating VNBC Lm: Live/Dead BacLight^TM^ staining, ethidium monoazide and propidium monoazide-stained real-time polymerase chain reaction (EMA- and PMA-PCR), direct viable count (DVC), 5-cyano-2,3-ditolyl tetrazolium chloride-4′,6-diamidino-2-phenylindole (CTC-DAPI) double staining, and carboxy-fluorescein diacetate (CDFA) staining. Of these five supplementary methods, the Live/Dead BacLight^TM^ staining and CFDA-DVC staining currently appear to be the most accurate for VBNC Lm enumeration. In addition, the impact of the VBNC state on the virulence of Lm is reviewed. Widespread use of these supplemental methods would provide supporting data to identify the conditions under which Lm can revert from its VBNC state into an actively multiplying state and help identify the environmental triggers that can cause Lm to become virulent. Highlights: Rationale for testing for all viable Listeria (Lm) is presented. Routine environmental sampling and plating methods may miss viable Lm cells. An overview and comparison of available VBNC testing methods is given. There is a need for resuscitation techniques to recover Lm from VBNC. A review of testing results for post VBNC virulence is compared

## 1. Introduction

*Listeria monocytogenes* (Lm) is a foodborne pathogen found in a variety of foods; outbreaks of listeriosis have been linked to the consumption of contaminated raw milk, ready-to-eat deli meats, cantaloupes, hot dogs, smoked fish, mushrooms, eggs, soft cheeses, frozen vegetables, packaged salads, ice cream, caramel apples, and bean sprouts [1]. The Foodborne Diseases Active Surveillance Network (FoodNet) currently monitors foodborne illness rates for about 15% of the United States population. In 2016 FoodNet confirmed 127 cases of listeriosis illnesses and 17 deaths within the surveillance populations, with a 97% hospitalization rate and a 13.4% mortality rate [2]. Using FoodNet data adjusted for geography, researchers calculated the annual number of listeriosis laboratory confirmed cases in the United States to be 808 [3]. Adjusting for underreporting and underdiagnosing, they estimated there are 1662 cases of listeriosis annually with a 95% hospitalization rate, about 1520 persons and a 15.9% death rate of 266 [3]. Due to the high mortality rate of listeriosis, as well as the risk of miscarriage in pregnant women from the disease, the Food Safety and Inspection Service (FSIS) of the United States Department of Agriculture (USDA) has instituted a zero-tolerance policy, where no viable Lm cells are permitted in any ready-to-eat (RTE) food products, regardless of whether or not the food supports the growth of Lm under its expected storage conditions [4]. In the instance of multiple *Listeria* positive food contact surface results, the food product lots are put on hold. A representative sample of 25 g of the food product lots are taken daily and any product lots on hold are not released until three consecutive days of negative results are obtained. The European Union also regulates Lm, including the requirement that Lm counts must be absent in 25 g samples of food intended for infants and foods for special medical purposes. All other foods must contain less than 100 CFU/g in RTE foods unable to support the growth of Lm [5]. The European Food Safety Authority (EFSA) reported 2502 cases of listeriosis in the EU in 2017, the highest infection rate was among persons over 64 years of age and in recent years, there has been an increasing trend of in the number of listeriosis cases [6].

In 2014, the USDA-FSIS published an updated guideline for the control of Lm in poultry and RTE products [4]. Any Lm contaminated RTE products are considered adulterated in inter-state commerce if they contain Lm or come into direct contact with a food contact surface that is contaminated with Lm. If RTE food products are exposed to the environment after a lethal processing step, they are required to adhere to one of three *Listeria* control alternatives to comply with USDA-FSIS guidelines. These alternatives include using a post-lethality treatment, antimicrobial agents or processes, or strict sanitation standards. With each of these alternatives, regular food product and environmental samples are taken to validate that the current Lm controls continue to be effective. Depending on the Lm control option chosen, testing may be required yearly, quarterly, monthly, every two weeks, or weekly. If a sample is found to be positive for Lm, intensified sampling is conducted. Increased sanitization efforts are recommended with increased sampling to verify that the source of Lm contamination has been eliminated. If issues with Lm control continue to persist, the food products may be put on hold and the establishments may be found in non-compliance [4].

Viable bacterial cells in a food processing plant are routinely exposed to stressful environments such as cleaning and sanitation operations, the depletion of available nutrients, or extended periods of desiccation. The loss of nutrients has been shown to be a trigger for viable bacterial cells such as Lm and other food pathogens to enter a viable but not culturable (VBNC) state [7,8]. In the VBNC state, bacteria cannot be cultured on standard plating agar but do maintain their cellular integrity with reduced metabolic activities, including ATP synthesis, expression of genes, and expression of mRNA [9,10,11,12]. Pathogenic microorganisms in the VBNC state may represent a potential food safety hazard because VBNC cells are not detected by routine, culture-based surveillance methods. Both the concepts of long-term persister cells [13] and of viable but non-culturable (VBNC) cells have been investigated in Lm. The VBNC state in Lm is typically caused by a reduction in available nutrients [14,15,16]. A change in temperature, low environmental pH, environmental salinity, chlorine stress, or exposure to sunlight may also play a role in triggering the induction of the VBNC state in Lm [15,17,18].

The inability of VBNC Lm cells to grow on traditional plating media can lead to false negatives during routine testing of products or food contact surfaces. It is also possible that given the right conditions the VBNC bacteria can resuscitate in vivo and regain virulence, thereby leading to infections, as demonstrated by Baffone et al. [19] while examining VBNC *Vibrio* using a rat ileal loop model. Thus, there may be a risk of Lm in the processing plant being undetected by traditional enrichment and plating techniques, being transferred to a RTE food and growing to a level that has a high probability of infecting a person when the food is consumed. Since regulators and the food industry are trying to minimize the risk of foodborne listeriosis, a new research focus is called for to understand the actual risks associated with *Listeria*’s VBNC state.

To create a nutrient-deprived environment in the laboratory, that induces the VBNC state in Lm, microcosm water consisting of filter-sterilized water, sterile deionized water, and water containing various levels of minerals are usually used [14,15]. Usually, Lm is first grown in a rich medium (such as brain-heart infusion broth), washed with microcosm water, then incubated in the microcosm water at 4 °C or 20 °C with gentle shaking. Regular plating on non-selective agars such as plate count agar [14,15] or blood agar [16] over a period of weeks is typically used to observe the decline in culturable cells. Once confirmed that the microcosm water contains no Lm capable of reproducing by traditional plating, the VBNC analysis is started.

To determine if Lm cells are completely non-viable or in a VBNC state, several laboratory analyses for viable cell detection have been developed. Each method attempts to assess some unique aspect of cell’s viability or metabolism. The five most widely used methods used are (1) Live/Dead BacLight^TM^ staining, (2) ethidium monoazide- and propidium monoazide-stained real-time polymerase chain reaction (EMA- and PMA-PCR), also known as viability PCR (v-PCR), (3) Direct Viable Count (DVC), (4) 5-cyano-2,3-ditolyl tetrazolium chloride—4′,6-diamidino-2-phenylindole (CTC-DAPI) double staining, and (5) carboxy-fluorescein diacetate (CDFA) staining although other more novel methods have been used to estimate the viability of Lm [20,21]. In this review, we will discuss the advantages and disadvantages of each of these five detection methods in some detail in the hope of encouraging additional studies that will help define the potential public health risk from VBNC Lm.

## 2. Methods Used to Identify Lm in the VBNC State

### 2.1. LIVE/DEAD BacLight^TM^ Staining

BacLight^TM^ is a differential staining method used to detect viable, non-viable and total bacteria cells present in a sample. The kit uses two nucleic acid-binding stains, SYTO 9 and propidium iodide (PI), contained in a dimethylsulfoxide solution (DSMO) [22]. SYTO 9 permeates the cell membrane of both viable and non-viable cells and stains these cells green. Propidium iodide only penetrates cells with compromised membranes (i.e., non-viable cells) and reduces the SYTO 9 stain, thereby staining the cells red. The green and/or red Lm cells are typically analyzed and enumerated using epifluorescence microscopy but can also be counted with flow cytometry [20,23,24,25].

When BacLight^TM^ staining was run in conjunction with flow cytometry, high correlations of live and dead Lm cell counts were found (r^2^ = 0.97 and r^2^ = 0.99 respectively) between ratios of 10% to 100% (living to dead cells) when compared to known control populations of live and dead cells [26]. BacLight^TM^ stained Lm cells under microscopy also gave a more accurate estimation of viable cell counts when compared to EMA/real-time PCR [27]. By using BacLight^TM^ staining in conjunction with flow cytometry and comparing it with direct viable counts, the membrane integrity of VBNC Lm populations can be demonstrated, even as plate-count cultivability was reduced to nearly 0% [20].

When the correlation between the percentage of metabolically active bacterial cells added to a sample was compared to the percentage of active cells measured by the BacLight^TM^ kit, a statistically significant correlation, r^2^ ≥ 0.98, was found for *A. hydrophila*, *B. subtilis*, *E. coli*, *P. aeruginosa*, and *S. epidermidis* [24]. When compared to 5-cyano-2,3-ditolyl tetrazolium chloride—4′,6-diamidino-2-phenylindole (CTC-DAPI) staining, BacLight^TM^ produced equal to or better accuracy in detecting viable and total counts of *E. coli* in several tests [22]. The accuracy of BacLight^TM^ is seen in a wide range of bacteria, and it is also effective in the analysis of Gram-positive pathogens such as Lm. BacLight^TM^ is commonly used to analyze Lm in biofilms [28,29] and to determine the effectiveness of antimicrobial treatments to specifically targeting cell enumeration in biofilms [29].

However, clear bimodal (green and red) staining is not always possible. Frequently, a gradient from dual staining is observed, resulting in some difficulty interpreting the results [30,31]. At the very least, mixed results warrant increased controls and verification with complementary methods; they might also suggest a gradient in cell viabilities. It also must be noted that cells with intact membranes are not always considered viable, and so an overestimation may occur [32]. This method is rapid, inexpensive, simple to perform, and is probably the most widely employed method for differentiating VBNC cells. It does, however, require an epifluorescent microscope or a flow cytometer. See Table 1 for a comparison of advantages and disadvantages of the various methods.

### 2.2. EMA- and PMA-Stained Real-Time PCR

Quantitative PCR (qPCR) targets the unique DNA sequences of foodborne pathogens such as Lm and amplifies these sequences to detectable levels. However, qPCR also has the potential to amplify intact DNA from dead Lm cells [33], and thus standard qPCR analysis may lead to an overestimation of viable cell counts [34]. This is especially problematic when attempting to detect VBNC Lm because VBNC bacteria do not grow using routine enrichment and plate count techniques. Due to these limitations, an environmental swab taken from a food contact surface and analyzed by qPCR could have viable Lm, VBNC Lm, and dead Lm cell DNA all being amplified. In the case of viable Lm and VBNC Lm, DNA being amplified by qPCR, the swab would correctly confirm a true positive. However, any amplified dead Lm cell DNA would confirm a false positive, leading to expensive, erroneous recommendations for corrective actions. Fortunately, there are additional techniques to minimize these false positives.

Ethidium monoazide (EMA) and propidium monoazide (PMA) are both DNA-binding agents used in combination with Real-Time Polymerase Chain Reaction (RT-PCR) to prevent the DNA of dead bacterial cells from being amplified. EMA penetrates damaged membranes of dead cells [35] and, when exposed to light, irreversibly binds to the dead cells’ DNA [36]. The EMA-bound cell DNA will not be amplified by subsequent PCR reactions, thus preventing false positives or over-estimation of viable cells [37,38], including Lm cells [35]. The exposure to light also inactivates any remaining free EMA in the sample, preventing subsequent binding to the DNA of viable cells during the DNA extraction step [39].

However, it is possible that EMA can penetrate viable cell membranes of certain bacterial species, including *Escherichia coli* O157:H7 [40] and Lm [37,41], which would result in too low an estimate of viable cells. To minimize this concern, Propidium monoazide (PMA) can be used as an alternative to EMA as a non-viable cell stain. Unlike EMA, PMA has not been shown to penetrate the membranes of living cells. This increased selectivity is possibly due to the higher molecular charge on PMA [37]. In conjunction with real-time PCR, PMA prevents the DNA of dead Lm cells from being amplified while allowing viable cell DNA (including VBNC cells) to be amplified, detected, and quantified, even in complex food matrixes [42].

Concerns have been recently raised with the use of PMA-based RT-PCR in determining viable cell counts. When analyzing heat-treated Lm, PMA-based RT-PCR discrimination between viable and dead cells depended on the extent of the heat treatment applied. Specifically, in the study by Lovdal et al. [43], Lm cells were heat treated at 60 °C for 6 min. When these cells were analyzed using PMA-based RT-PCR and viewed by microscopy following PMA-staining, it was determined the PMA-based RT-PCR did not prevent the DNA of membrane-compromised Lm cells from being amplified when high levels of heat-killed Lm cells were present with low levels of viable Lm cells [43]. In addition, the photo-activation step binding PMA to the non-viable cell DNA may be less effective at high microbial cell concentrations, as other cells may physically shadow the activating light from reaching all of the dead cells, thus leading to false positives [43]. Additionally, like other dyes, this method may overestimate the number of viable cells due to dead cells with intact membranes being incorrectly counted as viable [32]. Therefore, additional care should be used when evaluating viable cell numbers via PMA-based RT-PCR.

### 2.3. Direct Viable Count

The direct viable count (DVC) method was originally developed to enumerate bacteria in samples of seawater [44]. Its uses have expanded to the detection of VBNC cells of many bacterial species, including Lm [45]. In the DVC method, a limited level of nutrients (such as yeast extract) and an antibiotic that inhibits DNA replication are added to a water sample. To test for the VBNC state in Gram negative bacteria, nalidixic acid is usually used, but because it is not effective against Gram positive bacteria like Lm, an alternative antibiotic such as ciprofloxacin must be used [45]. Once the antibiotic and minimal nutrients are added, the water sample is incubated for 7 h at 37 °C. The antibiotic allows viable cells to begin the onset of cell growth and elongate in response to the yeast extract but prevents their cell division [44]. These elongated cells are then stained with a fluorescent dye and are subsequently directly counted with an epifluorescence microscope [45]. Any bacteria that have elongated to twice their normal cell length are considered to have been viable. The number of elongated cells can be compared to their corresponding plate counts to determine the number of cells in the VBNC state. The advantage of this method over other dye methods is that non-viable cells with intact membranes will not elongate. When analyzing microcosm water starved Lm cells for VBNC state, CTC-DAPI double staining and DVC resulted in the same average number of metabolically active Lm cells (10^6^ bacteria ml^−1^), with DVC analyzing the cell elongation of Lm and CTC-DAPI measuring cell respiration of Lm cells through CTC fluorescence [14]. Similar counts were also obtained between DVC and CTC-DAPI when analyzing NaCl levels as a physiochemical trigger for inducing VBNC state in Lm [15]. Highmore et al. [18] used a DVC variation with green-fluorescent protein producing Lm and pipemidic acid. They demonstrated that VBNC Lm remained above 10^6^ bacteria ml^−1^ when exposed to 50, 80, and 100 ppm chlorine, while traditional culturable plate counts were below the limit of detection [18]. Care must be used in analyzing DVC cells, as the microscopic determination of a doubling of cell length is quite subjective and prone to operator error.

### 2.4. CTC-DAPI Double Staining

CTC is a tetrazolium salt that is reduced to CTC-formazan by the electron transport system in actively respiring bacterial cells [46]. The CTC-formazan subsequently shows up as red/purple, fluorescent precipitant under epifluorescence microscopy. CTC has been shown to be an ideal stain due to its stability and being able to be detected at low levels because it shows a red/purple fluorescence [46]. DAPI passes through intact cell membranes, binding to the adenine-thymine (A-T) rich sequences of bacterial DNA [47], staining both living and dead cells blue. In using this double staining method, enumeration of total cell numbers, as well as respiring cell numbers, can be obtained simultaneously under epifluorescence microscopy.

### 2.5. CTC-DAPI

Staining used in conjunction with DVC is a common method for evaluating metabolic activity in VBNC bacterial cells and works well to detect VBNC Lm with reasonable accuracy [14,36]. CTC-DAPI staining is a precise method for analyzing viable cell counts within Lm biofilms after antimicrobial treatment and does not seem to overestimate viable cell counts when compared to EMA-qPCR [36]. With the CTC-formazan using cellular respiration to break down into a fluorescent dye, no dead cells with intact membranes should be stained. CTC-DAPI staining also gives comparable viable cell counts to DVC, with DVC measuring elongation of viable cells rather than their respiratory activity [48].

A similar stain, BacLight^TM^ RedoxSensor^TM^ Green may also be applied and analyzed using flow cytometry to confirm reductase activity of actively respiring cells [49]. BacLight^TM^ RedoxSensor^TM^ Green penetrates the cell membrane, and the reagent is reduced by enzymes in the electron transport chain of actively respiring cells, producing a green-fluorescent signal. Like the CTC-formazan, the BacLight^TM^ RedoxSensor^TM^ Green does not fluoresce in dead cells with intact membranes due to the lack of enzymatic reduction of the dye within the dead cells. This signal can then be analyzed, and actively respiring cells subsequently counted using a flow cytometer [49].

### 2.6. CFDA Stain

CFDA is a colorless fluorogenic ester that enters bacterial cells through diffusion. CFDA is then enzymatically cleaved by bacterial cell esterase enzymes to produce a fluorescent product, carboxyfluorescein (cF). The fluorescence is then analyzed by epifluorescence microscopy or through a flow cytometer; the intensity of the signal determines the viable cell count of the sample [36,50,51]. Due to the enzymatic breakdown of the fluorogenic ester in the dye, only viable cells with intact membranes are counted. Lm viable cell counts, analyzed by epifluorescence microscopy with CFDA, were significantly (*p* ≤ 0.003) higher when compared to plate counts when analyzing VBNC contamination of cheese [25]. When testing lake water samples using flow cytometry, CDFA produced significantly higher (*p* = 0.025) viable cell counts compared to control fluorescent labeling methods [52]. When used in conjunction with propidium iodide (PI), CFDA-based flow cytometry is an accurate method for determining viable cell counts. When analyzing the antimicrobial effects of oregano, thyme, and cinnamon essential oils on Lm, clear discrimination between viable cells and membrane-compromised cells was obtained using CFDA and PI [53]. Also, despite a strong reduction in plate counts of Lm, CFDA was retained in 40% of cinnamon oil-treated cells, suggesting that the Lm cells remained metabolically active [53]. CFDA and flow cytometry were used to evaluate the antimicrobial effectiveness of five essential oils against *L. innocua* [40]. The results suggested that several of the essential oils had permeated the bacterial cytoplasmic membrane and, based on the amount of CFDA fluorescence reduction caused by these oils, showed pronounced antimicrobial activity against the *L. innocua* [40].

CFDA results can also be compared to plating on non-selective media to determine VBNC counts of Lm when evaluating food storage conditions. When evaluating the formation of VBNC Lm on packaged hard cheeses, CFDA-based fluorescent microscopy showed a significant (*p* ≤ 0.003) difference in viable Lm counts when compared to direct plate counts over a period of 90 days regardless of storage conditions, suggesting that the Lm may have entered a VBNC state [25]. When methods of enumerating *Aeromonas hydrophila, Bacillus subtilis, Escherichia coli, Pseudomonas aeruginosa,* and *Staphylococcus epidermidis* were compared, no significant differences (*p* > 0.05) of metabolically active bacteria were obtained by both the BacLight^TM^ staining and CFDA staining in four of seven water samples when the differentially stained cells from both methods were compared using a flow cytometer [24].

## 3. Virulence

There are currently several methods for the accurate and precise detection and enumeration of metabolically active Lm. However, in addition to determining that these metabolically active Lm cells are VBNC cells waiting for the appropriate environmental conditions that will allow them to begin replication, we must also understand the expression of virulence in VBNC Lm. The pathogenic gene *hly* (which encodes for virulence protein Listeriolysin O) is an important virulence factor in Lm [16]. Its expression would suggest potential virulence of VBNC cells. Both *hly* and *inlA* expression continued after 27 days in VBNC Lm cultures starved in microcosm water [54]. Continued expression of the *hly* gene was demonstrated in VBNC Lm after starvation in microcosm water [16].

However, the expression of Lm virulence genes has yet to be unequivocally demonstrated to translate into Lm VBNC pathogenicity. Study results have been mixed in virulence models. VBNC Lm cells were tested in mouse models and were shown to be unable to colonize mouse spleens [16,48]. The indication here is that, because the Lm cells were unable to be resuscitated in the mouse model, the Lm cells had not regained their virulence through the inoculation of the host. Similarly, Recombination Activating 1 (*RAG1*)-deficient mice were injected with 10^7^ VBNC cells, sacrificed two weeks after injection, and their spleens harvested and plated on Brain Heart Infusion (BHI) plates. RAG1-deficient mice were chosen because RAG1 is required for somatic recombination of T-cell receptor (TCR) and Immunoglobulin (Ig) genes. Therefore, the absence of this protein resulted in immunodeficient mice [16]. Despite this immunodeficient host condition, no culturable Lm cells were recovered from the mouse spleens. When Lm was absent in the spleens of the mice originally infected with VBNC cells, it indicated that these VBNC cells did not resuscitate and thus remained avirulent. It can be hypothesized based on these results that, due to the inability for the Lm cells to reach the spleen, they may not be expressing the necessary proteins to cause infection.

In addition, VBNC Lm cells were not able to adhere to a HT-29 cell culture line. HT-29 was used as a human colon cancer cell line that can express characteristics of mature intestinal cells, giving an indication of microbial adhesion in the human gut [16,48]. Cappelier, et al. innoculated a HT-29 cell monolayer with culturable and VBNC Lm cells. While plaques were formed from three of the Lm strains in the culturable state, no strains of Lm in the VBNC state were able to form plaques on the HT-29 cell monolayer, thus classifying the VBNC cells as being in an avirulent state [48]. Similarly, Lindback, et al. inoculated HT-29 cell monolayers with culturable and VBNC Lm cells [16]. All 16 strains of Lm formed plaques in their culturable form. However, no strain in its VBNC state was able to form plaques, suggesting lack of virulence while in VBNC state [16].

In contrast to these negative findings of VBNC virulence for Lm, we will now discuss two studies with results that suggest VBNC Lm cells’ avirulence may be either transitory, or in the case of the second study, simply require the right environment to become virulent. The first study [55] replicated much of the work of the earlier work of Cappelier, et al. [48] who had demonstrated that starvation-induced VBNC Lm, which lacked virulence while VBNC, could be resuscitated; this resulted in the Lm cells returning to a state of cultivability and virulence. Using both a human colon cell line, HT-29, and a mouse model, these researchers [55] demonstrated that resuscitated VBNC Lm negatively affected both HT-29 (by causing plaques) and mice (by invading the spleen) in the same manner as their viable and cultivable counterparts. It appears that this difference in virulence results was due to the VBNC cells being first passed through embryonated eggs before infecting the HT-29 cells and being administered to the mice. These second set of results [55] suggest that the avirulence state of VBNC Lm may only be transitory. Again, these results need further support.

A second study used *Caenorhabditis elegans*, a nematode model [18]. VBNC Lm (induced by 200 ppm chlorine) were used to infect *C. elegans*. Not only did *C. elegans* ingest the VBNC Lm, but Lm was also found outside of the intestinal lumen. Furthermore, the exposure to VBNC Lm produced the reduced nematodes’ lifespan by a similar length as consuming viable, culturable Lm did. The authors suggest that this reduction in lifespan may be due to continued expression of Lm virulence genes during the VBNC state.

To determine how much of a risk VBNC Lm truly is, the likelihood of VBNC Lm resuscitation needs to be better understood. Therefore, the next research step is to look for effective ways of resuscitating the VBNC Lm into a culturable state. This is important for two reasons: (1) the ability to consistently resuscitate VBNC Lm would allow for its detection during routine in-plant environmental sampling and (2) research on resuscitation will hopefully shed light on under what conditions Lm VBNC are an increase public health food safety risk.

## 4. Conclusions

The study using the embryonic egg model to resuscitate VBNC Lm raises questions of a public health risk regarding the VBNC state of Lm [55]. Namely, why did this model work when others failed? There have been many attempts to resuscitate VBNC Lm using numerous environmental adjustments, adding the cells to nutrient rich medium, and directly injecting the VBNC cells into animal models. Some of these animal models, like mice, may have active immune systems in which the host’s phagocytosis would be expected to eliminate the VBNC Lm cells. The incubation times, temperatures, and growth media in the embryonic egg model did not vary greatly from other, unsuccessful protocols. However, in vitro models have the disadvantage of a short exposure duration and the medium is not completely representative of a mammalian gut. The nutrients, environment, and exposure time of an intestinal tract may differ from a cell culture model. In the successful model, the unique factor was the use of an embryonic egg for the resuscitation of the VBNC cells. Therefore, it may be hypothesized that there are either nutrients or other factors in the embryonic egg which are absent from other growth media previously examined or the required ratio of the nutrients is important. If the nutrient requirements for resuscitating VBNC Lm can be determined, then further questions can be investigated. For example, how likely is it that all or some of these nutrients can be found in the food processing environment? What necessary metabolic pathways do these nutrients activate so that VBNC Lm may resuscitate and subsequently regain virulence? What is the response of VBNC cells if introduced orally into an animal model rather than injection, and does that affect the ability to regain virulence?

Once we can answer these questions, we can better determine the health risks associated with the possibility of Lm resuscitating and subsequently expressing virulence in the environment or producing a food borne infection when consumed. We may also be able to develop standardized techniques for resuscitating VBNC Lm for food production plant and laboratory testing. We would adjust our testing methods so that a very small amount of Lm contamination could be detected, to meet the zero-tolerance requirements of the FDA. If the testing methods or the resuscitation requires many cells, then a potential infectious amount may be missed. Once we determine a proper testing method for VBNC Lm in the poultry processing plant, we may be able to adjust standard sanitization procedures to prevent the induction of Lm into VBNC state in the first place. If in fact the VBNC Lm cells can resuscitate and regain virulence in the environment or when consumed, this knowledge will better assist us in minimizing the risk of Lm associated foodborne illness.

At this early stage, we are not able to confidently estimate the public health risks associated with the VBNC state in Lm, but if these cells are not able to revert to an infectious state unless under preferred environmental and nutrient conditions, then this public health risk would be minimal. However, one cannot assume based on current testing that in vivo conditions would prevent VBNC Lm from causing foodborne illness, even if little evidence supports its ability to resuscitate after entering VBNC state. Therefore, until it is proven that VBNC Lm cannot cause illness in vivo, Lm in the VBNC should probably be included in the current USDA zero tolerance regulations. Current plate count detection focuses on live Lm in food and environmental samples, with negative results giving confirmation that the sanitizing/cleaning process is working correctly. The use of viability RT-PCR, DVC, CTC-DAPI double staining, BacLight^TM^ staining, and CFDA staining may allow for better detection/enumeration of total bacterial load and VBNC bacteria when compared to plate counts. Resuscitation using an embryonic egg model and the renewed expression of virulence in Lm has been proven possible [55], however questions about nutrient and environmental requirements remain. Further testing is needed to determine the probability that VBNC Lm can resuscitate and regain virulence outside a laboratory environment.

## Figures and Tables

**Table 1:** Summary of the advantages, disadvantages, and requirements for each of the enumeration methods.

	Advantages	Disadvantages	Mechanism	Indicates
Live/Dead BacLight^TM^ staining	Rapid, inexpensive. Stains cells green with intact cellular membrane, red compromised membrane. Highly correlated to live and dead cells	Gradient of or dual staining. Need epifluorescence microscope or flow cytometer to enumerate	Nucleic-acid binding stains	Membrane integrity
EMA- and PMA-PCR ^a^	Costly, requires trained technician	False positives	Nucleic-acid binding	Membrane integrity
DVC ^b^	Rapid, accurate with flow cytometry	Subjective interpretation of what is an elongated cell	Inhibits cell division	Cell growth
CTC-DAPI double staining ^c^	Does not overestimate as compared to EMA	Requires epifluorescence microscopy	Reduction of CTC by active electron transport system	Active electron transport system
CFDA ^d^	May produce higher viable cell counts. Similar results to BacLight^TM^	Requires epifluorescence microscopy or flow cytometer to enumerate	Enzymatic cleavage	Esterase activity produces a fluorescent product

^a^ Ethidium Monoazide- and Propidium Monoazide-stained real-time Polymerase Chain Reaction. ^b^ Ethidium monoazide (EMA) and propidium monoazide (PMA) are both DNA-binding agents used with quantitative PCR for Direct Viable Count. ^c^ 5-cyano-2,3-ditolyl tetrazolium chloride—4′,6-diamidino-2-phenylindole double staining. ^d^ carboxy-fluorescein diacetate staining.

## References

[1] Centers for Disease Control and Prevention (2020). *Listeria* Outbreaks. https://www.cdc.gov/listeria/outbreaks/index.html.

[2] Marder E.P., Cieslak P.R., Cronquist A.B., Dunn J., Lathrop S., Rabatsky-Ehr T., Ryan P., Smith K., Tobin-D’Angelo M., Vugia D.J. (2017). Incidence and Trends of Infections with Pathogens Transmitted Commonly through Food and the Effect of Increasing Use of Culture-Independent Diagnostic Tests on Surveillance—Foodborne Diseases Active Surveillance Network, 10 U.S. Sites, 2013–2016. MMWR Morb. Mortal. Wkly. Rep..

[3] Scallan E., Hoekstra R.M., Angulo F.J., Tauxe R.V., Widdowson M.-A., Roy S.L., Jones J.L., Griffin P.M. (2011). Foodborne illness acquired in the United States—Major pathogens. Emerg. Inf. Dis..

[4] United States Department of Agriculture—Food Safety and Inspection Service FSIS Compliance Guideline: Controlling Listeria Monocytogenes in Post-Lethality Exposed Ready-to-Eat Meat and Poultry Products. https://www.fsis.usda.gov/wps/portal/fsis/topics/regulatory-compliance/guidelines/2014-0001.

[5] European Commission (2005). Commission Regulation (EC) No 2073/2005 of 15 November 2005 on Microbiological Criteria for Foodstuffs.

[6] European Food Safety Authority and European Centre for Disease Prevention and Control Listeriosis—Annual Epidemiological Report. https://www.ecdc.europa.eu/en/publications-data/listeriosis-annual-epidemiological-report-2017.

[7] Kumar S., Ghosh A. (2019). Assessment of bacterial viability: A comprehensive review on recent advances and challenges. Microbiology.

[8] Zhao X., Zhong J., Wei C., Lin C.-W., Ding T. (2017). Current perspectives on viable but non-culturable state in foodborne pathogens. Front. Microbiol..

[9] Asakura H., Makino S., Takagi T., Kuri A., Kurazono T., Watarai M., Shirahata T. (2002). Passage in mice causes a change in the ability of *Salmonella enterica* serovar Oranienburg to survive NaCl osmotic stress: Resuscitation from the viable but non-culturable state. FEMS Microbiol. Lett..

[10] Dolezalova E., Lukes P. (2015). Membrane damage and active but nonculturable state in liquid cultures of *Escherichia coli* treated with an atmospheric pressure plasma jet. Bioelectrochemistry.

[11] Jia J., Chen Y., Jiang Y., Tang J., Yang L., Liang C., Jia Z., Zhao L. (2014). Visualized analysis of cellular fatty acid profiles of *Vibrio parahaemolyticus* strains under cold stress. FEMS Microbiol. Lett..

[12] Trinh N.T.T., Dumas E., Le Thanh M., Degraeve P., Amara C.B., Gharsallaoui A., Oulahal N. (2015). Effect of a Vietnamese *Cinnamomum cassia* essential oil and its major component *trans*-cinnamaldehyde on the cell viability, membrane integrity, membrane fluidity, and proton motive force of *Listeria innocua*. Can. J. Microbiol..

[13] Buchanan R.L., Gorris L.G.M., Hayman M.M., Jackson T.C., Whiting R.C. (2017). A review of *Listeria monocytogenes*: An update on outbreaks, virulence, dose-response, ecology, and risk assessments. Food Control.

[14] Besnard V., Federighi M., Cappelier J.M. (2000). Evidence of viable but non-culturable state in *Listeria monocytogenes* by direct viable count and CTC-DAPI double staining. Food Microbiol..

[15] Besnard V., Federighi M., Declerg E., Jugiau F., Cappelier J.M. (2002). Environmental and physico-chemical factors induce VBNC state in *Listeria monocytogenes*. Vet. Res..

[16] Lindback T., Rottenberg M.E., Roche S.M., Rorvik L.M. (2010). The ability to enter into an avirulent viable but non-culturable (VBNC) form is widespread among *Listeria monocytogenes* isolates from salmon, patients and environment. Vet. Res..

[17] Cunningham E., O’Byrne C., Oliver J.D. (2009). Effect of weak acids on *Listeria monocytogenes* survival: Evidence for a viable but nonculturable state in response to low pH. Food Control.

[18] Highmore C.J., Warner J.C., Rothwell S.D., Wilks S.A., Keevil C.W. (2018). Viable-but-nonculturable *Listeria monocytogenes* and *Salmonella enterica* Serovar Thompson induced by chlorine stress remain infection. mBio.

[19] Baffone W., Citterio B., Vittoria E., Casaroli A., Campana R., Falzano L., Donelli G. (2003). Retention of virulence in viable but non-culturable halophilic *Vibrio* spp.. Int. J. Food Microbiol..

[20] Gurresch A., Gerner W., Pin C., Wagner M., Hein I. (2016). Evidence of metabolically active but non-culturable *Listeria monocytogenes* in long-term growth at 10 °C. Res. Microbiol..

[21] Kapuscinski J. (1995). DAPI: A DNA-specific fluorescent probe. Biotech. Histochem..

[22] Boulos L., Prevost M., Benoit B., Coallier J., Desjardins R. (1999). LIVE/DEAD^®^BacLight(TM): Application of a new rapid staining method for direct enumeration of viable and total bacteria in drinking water. J. Microbiol. Methods.

[23] Allegra S., Berger F., Berthelot P., Grattard F., Pozzetto B., Riffard S. (2008). Use of flow cytometry to monitor *Legionella* viability. Appl. Environ. Microbiol..

[24] Hoefel D., Grooby W.L., Monis P.T., Andrews S., Saint C.P. (2003). Enumeration of water-borne bacteria using viability assays and flow cytometry: A comparison to culture-based techniques. J. Microbiol. Methods.

[25] Olszewska M.A., Panfil-Kuncewicz H., Laniewska-Trokenheim L. (2015). Detection of viable but nonculturable cells of *Listeria monocytogenes* with the use of direct epifluorescent filter technique. J. Food Saf..

[26] Stocks S.M. (2004). Mechanism and use of the commercially available viability stain, BacLight. Cytometry.

[27] Flekna G., Štefanič P., Wagner M., Smulders F.J., Možina S.S., Hein I. (2007). Insufficient differentiation of live and dead *Campylobacter jejuni* and *Listeria monocytogenes* cells by ethidium monoazide (EMA) compromises EMA/real-time PCR. Res. Microbiol..

[28] Giao M.S., Keevil C.W. (2014). *Listeria monocytogenes* can form biofilms in tap water and enter into the viable but non-cultivable state. Microb. Ecol..

[29] Lee S.H.I., Cappato L.P., Corassin C.H., Cruz A.G., Oliveira C.A.F. (2016). Effect of peracetic acid on biofilms formed by *Staphylococcus aureus* and *Listeria monocytogenes* isolated from dairy plants. J. Dairy Sci..

[30] Jacobsen C.N., Rasmussen J., Jakobsen M. (1997). Viability staining and flow cytometric detection of *Listeria monocytogenes*. J. Microbiol. Methods.

[31] Swarts A.J., Hastings J.W., Roberts R.F., von Holy A. (1998). Flow cytometry demonstrates bacteriocin-induced injury to *Listeria monocytogenes*. Curr. Microbiol..

[32] Joux F., Lebaron P. (2000). Use of fluorescent probes to assess physiological functions of bacteria at single cell level. Microb. Infect..

[33] McKillip J.L., Jaykus L.A., Drake M. (1999). Nucleic acid persistence in heat-killed *Escherichia coli* O157:H7 from contaminated skim milk. J. Food Prot..

[34] Reichert-Schwillinsky F., Pin C., Dzieciol M., Wagner M., Hein I. (2009). Stress- and growth rate-related differences between plate count and real-time PCR data during growth of *Listeria monocytogenes*. Appl. Environ. Microbiol..

[35] Rudi K., Naterstad K., Dromtorp S.M., Holo H. (2005). Detection of viable and dead *Listeria monocytogenes* on gouda-like cheeses by real-time PCR. Lett. Appl. Microbiol..

[36] Waring M.J. (1965). Complex formation between ethidium bromide and nucleic acids. J. Mol. Biol..

[37] Nocker A., Camper A.K. (2006). Selective removal of DNA from dead cells of mixed bacterial communities by use of ethidium monoazide. Appl. Environ. Microbiol..

[38] Nogva H.K., Dromtorp S.M., Nissen H., Rudi K. (2003). Ethidium monoazide for DNA-based differentiation of viable and dead bacteria by 5-nuclease PCR. BioTechniques.

[39] Rudi K., Moen B., Holck A.L. (2005). Use of Ethidium Monoazide and PCR in combination for quantification of viable and dead cells in complex samples. Appl. Environ. Microbiol..

[40] Nguefack J., Budde B.B., Jakobsen M. (2004). Five essential oils from aromatic plants of Cameroon: Their antibacterial activity and ability to permeabilize the cytoplasmic membrane of *Listeria innocua* examined by flow cytometry. Lett. Appl. Microbiol..

[41] Pan Y., Breidt F. (2007). Enumeration of viable *Listeria monocytogenes* cells by real-time PCR with propidium monoazide and ethidium monoazide in the presence of dead cells. Appl. Environ. Microbiol..

[42] Desneux J., Biscuit A., Picard S., Pourcher A.M. (2016). Fate of viable but non-culturable *Listeria monocytogenes* in pig manure microcosms. Front. Microbiol..

[43] Lovdal T., Hovda M.B., Bjorkblom B., Moller S.G. (2011). Propidium monoazide combined with real-time quantitative PCR underestimates heat-killed *Listeria innocua*. J. Microbiol. Methods.

[44] Kogure K., Simidu U., Taga N. (1979). A tentative direct microscopic method for counting living marine bacteria. Can. J. Microbiol..

[45] Besnard V., Federighi M., Cappelier J.M. (2000). Development of a direct viable count procedure for the investigation of VBNC state in *Listeria monocytogenes*. Lett. Appl. Microbiol..

[46] Rodriguez G.G., Phipps D., Ishiguro K. (1992). Use of a fluorescent redox probe for direct visualization of actively respiring bacteria. Appl. Environ. Microbiol..

[47] Jarvis N.A., O’Bryan C.A., Martin E.M., Ricke S.C., Johnson M.G., Crandall P.G. (2017). Further evidence of how unbuffered starvation at 4 °C influences *Listeria monocytogenes* EGD-e, HCC23, F2365, and Scott A. J. Food Protect..

[48] Cappelier J.M., Besnard V., Roche S., Garrec N., Zundel E., Velge P., Federighi M. (2005). Avirulence of viable but non-culturable *Listeria monocytogenes* cells demonstrated by in vitro and in vivo models. Vet. Res..

[49] Lahtinen S.J., Ahokoski H., Reinikainen J.P., Gueimonde M., Nurmi J., Ouwehand A.C., Salminen S.J. (2008). Degradation of 16S rRNA and attributes of viability of viable but nonculturable probiotic bacteria. Lett. Appl. Microbiol..

[50] Kramer B., Muranyi P. (2014). Effect of pulsed light on structural and physiological properties of *Listeria innocua* and *Escherichia coli*. J. Appl. Microbiol..

[51] Olszewska M.A., Zhao T., Doyle M.P. (2016). Inactivation and induction of sublethal injury of *Listeria monocytogenes* in biofilm treated with various sanitizers. Food Control.

[52] Porter J., Diaper J., Edwards C. (1995). Direct measurements of natural planktonic bacterial community viability by flow cytometry. Appl. Environ. Microbiol..

[53] Paparella A., Taccogna L., Aguzzi I., Chaves-Lopez C., Serio A., Marsilio F., Suzzi G. (2008). Flow cytometric assessment of the antimicrobial activity of essential oils against *Listeria monocytogenes*. Food Control.

[54] Zolfaghari M., Rezaei M., Mobarez A.M., Moghaddam M.F., Hosseini H., Khezri M. (2019). Virulence genes expression in viable but non-culturable state of *Listeria monocytogenes* in fish meat. Food Sci. Technol. Int..

[55] Cappelier J.M., Besnard V., Roche S., Velge P., Federighi M. (2007). Avirulent viable but non culturable cells of *Listeria monocytogenes* need the presence of an embryo to be recovered in egg yolk and regain virulence after recovery. Vet. Res..

